# PEGASUS: the Design of an Intervention to Facilitate Shared Decision-making in Breast Reconstruction

**DOI:** 10.1007/s13187-019-01656-6

**Published:** 2020-01-28

**Authors:** A. Clarke, N. Paraskeva, P. White, P. Tollow, E. Hansen, D. Harcourt

**Affiliations:** 1 Centre for Appearance Research, University of the West of England, Frenchay Campus, Cold Harbour Lane, Bristol, BS16 1QY UK; 2 Royal Free London NHS Foundation Trust, Pond Street, London, NW3 2QG UK

**Keywords:** PEGASUS, Behaviour change, COM-B, Shared decision-making, Breast reconstruction

## Abstract

Studies have found varying levels of satisfaction after breast reconstruction surgery with a substantial group of patients reporting some level of regret about their decision. The variable outcomes reported by women undergoing breast reconstruction surgery suggest a role for improved pre-operative communication and shared decision-making (SDM) between patient and health professional. Pragmatic approaches such as decision aids have been evaluated, but the aim of the Patient Expectations and Goals Assisting Shared Understanding of Surgery (PEGASUS) intervention is to facilitate closer interaction between the patient and clinical team. PEGASUS is a standardised two-stage process, in which patients’ goals are first elicited, ranked in importance and recorded before being used to frame discussion and decision-making with the surgeon managing care. Following the Medical Research Council (MRC) model, feasibility and acceptability studies have already been reported and a 4-year multicentre randomised controlled trial of 180 participants is underway, (completion 2020). This paper therefore focuses on the design of the intervention itself, in line with recent advice that interventions, in comparison with evaluations, commonly lack a theoretical base and are often under reported. We report a retrospective application of the Capability, Opportunity, Motivation-Behaviour (COM-B) model to provide explicit detail of each step in the intervention design. This is intended to facilitate replication by other clinicians and to provide systematic guidance for others wishing to develop PEGASUS as a strategy for implementing SDM in other clinical populations. Trial Registration: ISRCTN 18000391 (DOI 10.1186/ISRCTN18000391) 27/01/2016.

## Introduction

### Breast Reconstruction

Over 5000 women each year in the UK undergo breast reconstruction after mastectomy, with international efforts to increase the uptake of reconstruction including good practice guidelines (UK) and the implementation of the Breast Cancer Patient Education Act (2016). In the USA. however, uptake remains variable in both settings [1, 2]. Offered as an immediate adjunct to mastectomy or as a delayed procedure, breast reconstruction aims to restore the aesthetics of the breast mound rather than breast function and as such, is aimed at the restoration of quality of life and body image. Relevant patient reported outcomes such as satisfaction with shape, symmetry, sensation and impact on lifestyle are therefore subjective and impacted by individual differences. For example, satisfaction with the appearance of the breast is influenced as much by the level of individual investment in appearance as by the (vertical) symmetry of the breast [3]. There is also substantial evidence from the wider field of body image research that objective appearance is not a predictor of psychosocial outcomes [4].

Perhaps not surprisingly therefore, studies have found varying levels of satisfaction after surgery with a substantial group of patients reporting some level of regret about their decision [5]. The National Mastectomy and Breast Reconstruction Audit [1] reported 1/3 of immediate and 22% of delayed reconstruction patients remained disappointed with outcome at 12 months, with satisfaction with sexual function as low as 52% for women having immediate breast reconstruction when assessed at 18 months. Not only are a significant number of patients disappointed with the aesthetics of the breast, e.g. visibility of scarring [6] but with impact on their quality of life, e.g. feelings of femininity and sexual activity. The fact that patient expectations may not be met is of concern given the prevailing recommendation that all women should be offered the option of breast reconstruction and the efforts to increase its uptake.

### Information and Decision-making

Good information about the procedure is essential to underpin a complex decision-making process involving discussion of both surgical and individual psychosocial outcomes. Breast reconstruction is an umbrella term for a group of techniques ranging from implant-based reconstruction to the use of multitissue allografts in which tissue is removed from one area of the body (e.g. the abdomen) and grafted with its blood supply to the breast area. The process of breast reconstruction may be carried out in stages and may include creation of new nipple and symmetrisation of the contralateral breast. According to the patient’s history and lifestyle, one technique may be more favourable than another including no reconstruction at all.

However, there is an important distinction to be made within health care, between *information provision per se*, which may be very detailed and thorough, and *the process of how the information is used to make a decision*, which will be variable [7]. NHS England defines shared decision-making as a process which ‘ensures that individuals are supported to make decisions that are right for them. It is a collaborative process through which a clinician supports a patient to reach a decision about their treatment. The conversation brings together: the clinician’s expertise, such as treatment options, evidence, risks and benefits and what the patient knows best: their preferences, personal circumstances, goals, values and beliefs’.

Increasingly, many, but not all, patients want information about health care choices and to be fully involved in the decision-making process. How this shared decision-making process is facilitated varies from a simple set of recommended steps [8, 9] to the use of decision aids and decision coaching [10]. For example, a recent systematic review evaluated 23 different decision aids designed to assist women in their choice of treatment for early-stage breast cancer, reporting no differences between delivery via Internet, audio, booklet or video modalities [11]. However, decision aids are commonly designed for self-completion and whilst providing information, do not directly facilitate the reciprocal interchange of information via a conversation between surgeon and patient as indicated in the NHS England explanation (above). Notwithstanding the disagreement about how best to define and implement shared decision-making, the considerable evidence of a positive relationship between patient involvement in the decision-making process and high levels of satisfaction has driven the move to embed shared decision-making throughout the health care setting in the UK [12, 13]. In line with these developments, shared decision-making has potential utility for women considering breast reconstruction, with the following factors of particular relevance:

1. Satisfaction with outcome is the key target for change
2. Shared decision-making is associated with positive outcomes
3. Both aesthetic and individual psychosocial goals must to be understood in assessing expectations of outcome
4. Breast reconstruction involves complex decision-making

We, therefore, designed an intervention (Patient Expectations, and Goals Assisting Shared Understanding of Surgery (PEGASUS)) as a response to the National Mastectomy and Breast Reconstruction Audit [1] with the goal of improving outcomes by developing a shared decision-making model to manage expectations and underpin surgical planning.

### Design and Development of Interventions

The development and evaluation of interventions to change health behaviours is a topic receiving considerable current attention, with acknowledgement that these are often multicomponent processes involving complex interaction between patients, health professionals and contextual factors [14]. Various models have been suggested to support evidence based systematic approaches to their development. For example, the MRC model for development and evaluation of complex interventions [15–17] is widely used but has been criticised for its focus on the evaluation process rather than the specific content of an intervention; a simple and pragmatic series of six steps with more detailed recommendations on content design (Six Steps in Quality Intervention Design 6SquID) has been suggested as an alternative [14]. However, the most comprehensive approach to development of interventions to support behaviour change is the COM-B model [18, 19]. This model uses an associated taxonomy of behaviour change techniques and has got a considerable body of work supporting its use in clinical settings (http://www.bct-taxonomy.com/interventions). Both 6SquID and COM-B address the question of which potential targets for change are the most easily measured, are valid in addressing the underlying problem and which behaviour change techniques have the potential for greatest impact. COM-B also offers the potential to clarify the content of the intervention very clearly so that the process of shared decision-making is made explicit.

In developing our intervention PEGASUS, we followed the MRC model for complex interventions, developing the content systematically, refining and testing in practice, collecting feedback from patients and health professionals as per the model, before carrying out a feasibility and acceptability study [20] prior to a randomised controlled trial (RCT). However, the considerable current interest in the approach following this first publication requires a systematic and more thorough description of the development of PEGASUS in order to allow other researchers to develop modifications for other patient groups. We note the precedent of using COM-B retrospectively in coding BCTTv1 as part of the Theories and Techniques of Behaviour Change Project (www.ucl.ac.uk/health-psychology/research/TaT) and have therefore used COM-B in a retrospective application to describe the development of PEGASUS as clearly as possible, to identify any potential additions and to facilitate further development.

The COM-B model proposes that behaviour is determined by three components: for any behaviour to be enacted, an individual needs to be motivated to engage in the behaviour, have the opportunity to do so and be capable of doing so. Each of these sources of behaviour is further divided to distinguish between physical and social opportunity, automatic and reflective motivation and physical and psychological capability.

These sources of behaviour form the hub of the Behaviour Change Wheel (BCW) [19], forming an inner ring. There are nine intervention functions (categories of inputs aiming to change behaviour) forming the middle ring. These are education, persuasion, incentivisation, coercion, training, enablement, modelling, environmental restructuring and restrictions. Seven policy categories form the final ring: service provision, regulation, fiscal measures, guidelines, environmental/social planning, communication/marketing and legislation. Having identified the problem behaviour and the target behaviours that support and maintain it, intervention functions and policy categories are selected which are thought to have the best potential utility for achieving change.

## Method

The steps taken to design PEGASUS can be summarised using the BCW, in eight steps:

First, the problem was identified, specified and defined as a behaviour that could be measured. Second, a target behaviour was chosen which had the highest potential for impacting on the problem. This was then specified, including how and when this behaviour was enacted, who would do it and where it would take place. Identifying what needed to change depended on careful assessment of current practice, discussion with the staff involved and the existing and potential barriers. The BCW was then used to identify the intervention functions (specific behavioural inputs) and potential policy categories with the greatest utility in achieving change. In the last two steps, specific behaviour change techniques were identified and fully described and the design and delivery of PEGASUS was finalised for testing.

## Results

### Identify, Define and Specify the Problem Behaviour

The problem behaviour and the key outcome measure in this study is ‘dissatisfaction with outcome of breast reconstruction as expressed on the Breast-Q’ [21]. This definition was achieved using the National Mastectomy and Breast Reconstruction Audit [1] as a data source. The national audit recruited 18,216 individuals from all NHS sites providing a service, over a 15-month period. Outcome data included clinician reported and patient reported outcomes using standardised measures. It therefore represents a substantial and robust data source from which to identify the problem. This decision was made in discussion with three clinical psychologists working with the client group and instrumental in the development of PEGASUS as a response to the audit findings.

### Select Target Behaviours

Target behaviours are those activities that are selected for change in order to impact on the problem behaviour. (For example, if the problem behaviour is smoking, then target behaviours might include avoidance of venues where people are smoking). Target behaviours need to be both observable and measurable. For this reason, despite noting its importance, other researchers [22] have avoided a focus on shared decision-making because of the problems with definition and measurement. (Shared decision-making involves multiple behaviours in both the patient and clinician groups). However, there was a strong consensus among the psychologists working within breast care that shared decision-making had both high potential utility in impacting patient satisfaction and also high face validity for both patients and health professionals participating in the intervention. Therefore shared decision-making was operationalised by breaking it down into a series of constituent behaviours which can be said to support the process (Table 1). These behaviours are not intended as an exhaustive list, or as a complete definition of shared decision-making, but as a summary of behaviours that are potentially important, observable and achievable and can be said to support a shared decision-making process in breast reconstruction.

**Table 1:** Determinants of patient satisfaction after breast reconstruction and potential target behaviours underpinning shared decision-making

Determinants of patient satisfaction after breast reconstruction (summarised from National Audit 2011)	Potential target behaviours
Surgical and aesthetic • Post-op appearance of the breast including scarring (of the breast and donor site), symmetry, appearance (clothed and unclothed) • Softness of the breast • Pain, swelling, sensation, arm and shoulder pain (in particular following Lattissimus dorsi procedures) abdominal and lower back pain and discomfort (in particular after DIEP procedures) Psychosocial • Emotional wellbeing including confidence in social settings, feeling feminine, normal, attractive, of equal value to other women • Sexual wellbeing including: feeling sexually attractive (clothed and unclothed), comfortable in sexual activity, satisfied with sexual life • Lifestyle wellbeing including for lattissimus dorsi: back and shoulder pain, difficulty in lifting objects, raising arms above head, doing up zippers, hair styling: repeated activities such as throwing balls, playing tennis: for DIEP, sitting up, ordinary day-to-day activities such as making bed, pulling in the abdomen, lower back pain Experience of care process • Including quality and amount of information, involvement in decisions, understanding of what the patient wanted, sensitivity of staff, availability, reassurance	Explain PEGASUS approach to shared decision-making • Role of health professional • Role of patient Elicit information from patient: • The cancer journey so far • Experience of care • Expectations of outcome of breast reconstruction (aesthetic, psychosocial and process) Introduce expert information to discuss and modify expectations where appropriate Elicit, rate importance and record patient goals • Aesthetic and surgical • Psychosocial and lifestyle Use PEGASUS sheet to record: • Exchange information • Inform and frame surgical planning • Discuss and modify expectations if appropriate • Prompt and use as reminder at home and with relatives • Inform final decision about surgery

The COM-B model suggests selecting only those behaviours that are the easiest to measure and have the greatest potential utility for achieving change; however, since all the behaviours above were considered achievable and likely to have impact, all were included in the intervention.

### Specify Target Behaviours

The third stage in the process of delivering COM-B requires the specification of the target behaviours; i.e. exactly what is being done, how often, when and by whom. PEGASUS was constructed as a two-stage process involving a first session with a coach (either a psychologist or breast care nurse according to current practice in the clinic) and a second session with the surgeon (Table 2).

**Table 2:** Specification of target behaviours

Target behaviour	Who needs to do the behaviour?	What do they have to do differently?	When do they do it?	Where do they do it?	How often do they do it?	With whom do they do it?
Elicit patient experience of cancer journey so far	Coach	Ask questions and reflect back before providing information	At the first meeting	In the breast clinic	Once	Patient
Provide information	Coach	Differentiate information about surgical, psychosocial and process outcomes	At the first meeting	In the breast clinic	Once	Patient
Explain shared decision making	Coach	Outline the role of the health professional and the role of the patient	At the first meeting	In the breast clinic	Once	Patient
Elicit patient understanding and expectations	Coach	Differentiate surgical (aesthetic) and psychosocial expectations	At the first meeting	In the breast clinic	Once	Patient
Record and rank goals	Coach	Write down patient goals differentiating surgical and psychosocial goals	At the first meeting	In the breast clinic	Once	Patient
		Rate importance of each goal from 0 to 10				
Share information with patient	Coach	Provide copy of the written record	At the first meeting	In the breast clinic	Once	Patient
Discuss the surgical decision	Surgeon	Use the written record to frame discussion of potential surgery	At the second meeting	In the breast clinic	Once	Patient
Set expectations	Surgeon	Identify which patient goals are most directly to be impacted by surgery (aesthetic) and which least (psychosocial)	At the second meeting	In the breast clinic	Once	Patient
		Rate probability of achieving surgical goals	At the second meeting	In the breast clinic	Once	Patient
Plan next steps	Surgeon	Share record of consultation with patient	At the second meeting	In the breast clinic	Once	Patient
		Jointly agree a treatment plan	At the second meeting	In the breast clinic	Once	Patient

### Identify What Needs to Change via COM-B Analysis

The next step involves an assessment of which of the sources of behaviour (capability, opportunity and motivation) need to change for each group of participants. For shared decision-making, this means the patient and the health professional involved. The psychologists working on the development of the intervention made this selection based on their experience of current clinical practice in one major centre. Given the way in which usual care was organised, patients and surgeons already had the *opportunity* to share decision-making in that the care pathway was already set up with clinic appointments arranged. Both parties were *physically capable* of carrying out the intervention but input to modify *psychological capability* was a clear priority. Thus, patients needed to have appropriate information and to be able to distinguish between surgical outcomes and psychosocial and short and long-term goals. Surgeons needed to understand and remember the process, modify their usual way of working and make plans for dealing with competing priorities. Both coach and surgeon needed to trouble shoot by developing if …then rules (e.g. *if* the patient is finding it hard to follow the process *then* I will use another patient example; *if* the photocopier is broken *then* I can copy the record after the clinic and organise sending to the patient). Both aspects of motivation are important. *Automatic motivation* is important to establish new habits and routines so that the intervention is completed systematically. *Reflective motivation*, as here, is often a critical component of complex interventions. In this setting both surgeon and patient are being asked to reflect on specific individual goals for surgery. Each has the task of informing the other about their perspective, the patient explaining why and how they believe surgery can help them, the surgeon reflecting on what is achievable (e.g. a particular size of breast), or what is less predictable or outside their control (feeling more feminine), i.e. modifying expectations where needed. The final shared decision depends on reviewing the importance of the goals the patient has identified and using a written record of the process to assist a surgical plan.

### Identify Intervention Functions

Once the relevant sources of behaviour have been identified, the behaviour change wheel is used to select the intervention functions most relevant to achieve change.

Education is the first important concept for this intervention. Patients considering breast reconstruction are routinely provided with information, but for shared decision-making to be effective, *how the information is used to support the process* must be understood. Therefore, health professionals needed to understand the PEGASUS model and how to embed it within their practice. Persuasion was necessary to change routine health professional practice. Training was important, with modelling from another team member (either via sitting in for the clinic or via video taped session to demonstrate the process). Although it is possible that a surgeon could carry out the whole PEGASUS process in one rather than two stages, enabling the final decision-making process to be built on an earlier explorative session with a coach provided a way of promoting shared decision-making within the whole clinic team. Therefore the final intervention functions selected by the psychologists developing the intervention were: education, persuasion, training, modelling and enablement.

### Identify Policy Categories

No policy categories were selected during the early development of this intervention. The most likely candidate according to the behaviour change wheel could have been service provision, but since patients in this particular centre were already offered a psychology appointment as part of the routine assessment for breast reconstruction, no service changes were required; indeed, the intervention was deliberately designed so that no additional costs would be incurred during the development phase. In other settings, changes in service provision (e.g. an additional session with a coach) should be considered.

### Identify Behaviour Change Techniques

Specific behaviour change techniques (BCTs) to deliver the intervention functions were identified using the behaviour change techniques taxonomy (BCTTv1) which classifies 93 discrete behaviour change techniques in 19 different categories. Our example script (for stage one), which had been role played and videoed to support training, was analysed to identify all BCTs by cross-reference to the taxonomy.

Table 3 summarises all the BCTs observed. Of these, those selected as essential components of PEGASUS were 1.2 goal setting (behaviour), 1.2 problem solving, 1.3 goal setting (outcome), 1.4 action planning, 1.7 review outcome goals, 4.2 information about antecedents, 4.3 re-attribution, 5.1 information about health consequences, 5.3 information about social and environmental consequences, 5.6 information about emotional consequences, 6.2 social comparison and 9.2 pros and cons. Additional BCTs were observed but were not considered an essential component of the intervention; they may or may not be used as part of any consultation depending on individual presentation. Potentially useful additions to PEGASUS identified in the taxonomy included: 1.9 commitment, ask the patient to use an ‘I will’ statement to affirm or reaffirm a strong commitment (i.e. I will bring the form to my next appointment)’ and 3.2 social support, encourage the patient to consider what help they might need.

**Table 3:** Analysis of the behaviour change techniques being used within the PEGASUS intervention

No.	Behaviour change techniques	Session 1: coach	Session 2: surgeon
		Target behaviour: elicit and agree a written record of the factors influencing the decision to have BR	Target behaviour: use the PEGASUS written record to inform and discuss options about BR and to make (and record ) the final decision
	Label	Examples	Examples
1.1	Goal setting (behaviour)	Generate discussion with the patient of their current understanding and the expectations that underpin their decision to have BR surgery with specific focus on both expectations of physical and psychosocial factors	Reintroduce the PEGASUS sheet as the basis for discussing options and sharing information about surgery Ask whether there have been any changes since previous consultation Explain that the purpose of the session is to make a final decision about surgery and plan next steps
1.2	Problem solving	Identify specific factors that are important for them including expectations of physical appearance and psychosocial consequences including wearing a prosthesis Prompt the patient to identify barriers preventing them from making a decision such as weighing up the pros and cons, need for more information etc.	Ask if the patient requires more information Discuss the different options available on the basis of the history and clinical examination, together with the expectations and preferences of the patient set out on the PEGASUS form Offer advice and modify expectations as appropriate
1.3	Goal setting (outcome)	Summarise in writing and rate importance of each specific physical and psychosocial goal for the individual on the PEGASUS form	Summarise the decision with the patient Record the decision
1.4	Action planning	Encourage a plan to review the PEGASUS form with friends or family and revise if needed. Set a time for doing so Ask patient to take PEGASUS form to appointments with the surgeon Show the PEGASUS form to the surgeon	Encourage a plan to review the decision with friends or family and revise if needed. Set a time for doing so. Offer follow-up appointment or Book patient for next step towards surgery/discharge
1.5	Review behaviour goal(s)	Review goals if patient is seen for a second appointment (NB: most are seen only once)	Review decision if patient is seen for a second appointment (NB: most are seen only once)
1.7	Review outcome goal(s)		Review the factors listed on the PEGASUS sheet as determined in session 1 Consider modifying surgical decision in light of the importance of goals ranked on PEGASUS form, e.g. if physical activities using the arms are rated as highly important (such as tennis) ensure that patient is aware of implications of one BR procedure v another
1.9^a^	Commitment	Ask the person to use an ‘I will’ statement to affirm or reaffirm a strong commitment (i.e. ‘I will bring the form to my next appointment and show it to the surgeon’)	Ask the person to use an ‘I will’ statement to affirm or reaffirm a strong commitment (i.e. ‘I will give up smoking before being added to surgical list’)
2.4	Self-monitoring of outcome(s) of behaviour	Encourage the patient to record any additional factors that occur to them or that emerge in discussion with others Encourage the patient to look at the PEGASUS sheet again before the appointment with surgeon to ensure that all factors are still relevant and included	
3.1	Social support (unspecified)	Consider introduction to support groups and advise sources of support where relevant	Support attendance at support group where relevant
3.2^a^	Social support (practical)	Encourage the patient to consider what help the individual might need (e.g. opportunity to talk things through with other people, coming with them to an appointment) and how this might be provided	If a decision is proving difficult, encourage the patient to consider what help the individual might need (e.g. opportunity to talk things through with other people, coming with them to an appointment) and how this might be provided
3.3	Social support (emotional)	Ask the patient to consider taking a partner or friend with them to their surgical appointment	
4.2	Information about antecedents	Review previous decisions and personal resources used to come to difficult decisions in general	
4.3	Re-attribution	For example, patient using incorrect information to bias decision-making, e.g. If the person attributes the need for BR to full recovery from cancer, reintroduce the idea that cancer is treated differently and many women choose not to have BR without compromising recovery	For example, patient using incorrect information to bias decision-making, e.g. If the person attributes the need for BR to full recovery from cancer, reintroduce the idea that cancer is treated differently and many women choose not to have BR without compromising recovery
5.1	Information about health consequences	Encourage patient to make full use of all information resources Direct patient to appropriate sources of information Consider the use of a decision aid	Encourage patient to make full use of all information resources Direct patient to appropriate sources of information Discuss the outcome of using a decision aid if appropriate
5.3	Information about social and environmental consequences	Explain that satisfaction with decision-making is higher if people carefully consider all the factors that are important to them and the probability that these can be achieved	Explain that the consequences re cancer are the same whether BR is undertaken or not Encourage patient to make the decision for her that is consistent with her values and beliefs set out in PEGASUS
5.4	Monitoring of emotional consequences	Encourage patient to reflect on how they would feel in the future if they take time to consider all the reasons that BR might be important for them and the likely outcomes	Encourage patient to reflect on how they would feel in the future if they make a decision which is concordant with the preferences and expectations they have about BR
5.5	Anticipated regret	Ask the person to assess the degree of regret they will feel if they believe that they did not consider all the factors important for them in considering BR	Ask the person to assess the degree of regret they will feel if they believe that they made a decision that was inconsistent with the all the factors important for them in considering BR or failed to make a decision
5.6	Information about emotional consequences	Explain that physical changes do not necessarily result in psychosocial changes (e.g. ‘feel a whole woman’). Taking time to elicit all the factors that are important for an individual is an important part of the process of coming to a decision and likely to result in higher satisfaction with the final decision made	Explain that physical changes do not necessarily result in psychosocial changes (e.g. ‘feel a whole woman’). Making a decision which is concordant with the preferences and expectations they have about BR is likely to result in higher satisfaction with the final decision made whether or not the final choice is to have BR or not
6.1	Demonstration of the behaviour	Use video example or case histories to model how different women have different priorities in considering the factors important in considering BR	Use video example or case histories to model women making different decisions about BR
6.2	Social comparison	Draw attention to the fact that women have different priorities and expectations in coming to a decision about whether to have BR	Draw attention to the fact that women make different decisions about whether or not to have BR
6.3	Information about others’ approval	Use case histories to illustrate the reaction of other people to the factors people consider important in weighing up whether to have BR	Use case histories to illustrate the reaction of other people to different decisions that patients make (NB: partners may be relieved that further surgery is being avoided)
7.1	Prompts/cues	Discuss the time and place that the patient may choose to review the factors important in decisions about BR and ? discuss with another person	Consider making a further appointment f the patient is unable to reach a decision
9.1	Credible source	Use of examples such as Angelina Jolie but discuss in terms of the factors she will have considered in thinking about surgery, not the decision itself	Use of examples such as Angelina Jolie but point out that other people make different choices with which they are equally satisfied
9.2	Pros and cons	See section 5 NB: this is pros and cons of making the decision not pros and cons of having surgery	Use the weightings for each goal on the PEGASUS sheet to help the patient identify the importance of each outcome for her and the probability of achieving this via surgery
9.3	Comparative imagining of future outcomes	Prompt the person to imagine and compare likely or possible outcomes following a full assessment of their individual preferences for BR for them rather than generic guidance	Prompt the person to imagine and compare likely or possible outcomes having made a fully informed decision based on their individual preferences versus generic guidance
10.9	Self-reward	Encourage to reward self with material (e.g. new clothes) or other valued objects if and only if they have clearly progressed decision-making, e.g. discussed with partner	
11.2	Reduce negative emotions^a^	Advise on the use of stress management skills, e.g. relaxation, pacing of activities	
11.3	Conserving mental resources	Advise to consider decision-making at certain times in the week rather than constant rumination	
12. Antecedents			
12.1	Restructuring the physical environment	Encourage patient to think about a quiet time when they will not be interrupted in order to discuss decision-making with partner/friend (e.g. arrange baby sitter, or ask person specifically to come to discuss this issue)	
12.6	Body changes	Prompt healthy eating, relaxation and exercise as alternative ways of improving positive self concept and confidence in ability to make decisions	
13.1	Identification of self as role model	Inform the person that taking time to consider all the factors important to them in making a decision creates a powerful role model for others in the family and friends who may face the same challenge	Inform the person that making a decision either for or against surgery creates a powerful role model for others in the family and friends who may face the same challenge, regardless of whether BR is chosen
13.2	Framing/reframing	Suggest that the person might think of the process of eliciting more information about BR as reducing stress about future decision-making rather than increasing it	Suggest that the person might think of making a decision about BR as reducing stress rather than increasing it, whatever the choice made
13.3	Incompatible beliefs	Draw attention to any difficult decisions made in the past, e.g. change of job, relationship etc.	
15.1	Verbal persuasion about capability	Encourage the person that they can successfully identify and weigh up the important factors in making this decision as they did in all the other stages of the cancer journey	Encourage the patient to believe that they can make a decision that is right for them provided they think about all the reasons that underpin it and act in accordance with these factors
15.2	Mental rehearsal of successful performance	Advise to imagine how they will feel once the decision has been fully explored and summarised	Advise to imagine how they will feel once the decision has been made
15.3	Focus on past success	Advise to describe or list the occasions on which the person has had to inform themselves about making a difficult decision	Encourage patient to consider previous occasions when they made a difficult decision and the factors that assisted them

^a^BCTs with potential utility in future developments of PEGASUS

### Design of the Intervention Including Setting, Frequency and Staff Training

PEGASUS has been delivered as part of an existing current clinical pathway [20]. Table 4 illustrates the final intervention design, set out as a summary of actions for those delivering PEGASUS.

**Table 4:** A COM-B theory-based design of an intervention designed to support shared decision-making for women considering breast reconstruction (PEGASUS)

Step	Action
Stage 1: the meeting between the patient and the trained PEGASUS coach; the key components^a^	
Step 1	Introduce the PEGASUS intervention. 1.1. What does PEGASUS stand for? 1.2. Audit findings; unmet expectations.
Step 2	‘You know you’—you are the expert on your life.
Step 3	Brief overview of the patient’s pathway.
Step 4	Explain how this session will work. 4.1. Check patient understands the purpose of the session.
Step 5	Elicit the patient’s surgical goals and write them down on the PEGASUS sheet.
Step 6	Explain the rating system and ask the patient to rate each surgical goal (from 0 -10) in regards of its importance to them. 6.1. Rating helps everyone see which goals are the most important.
Step 7	Elicit the patient’s psychosocial (lifestyle) goals and write them down on the PEGASUS sheet.
Step 8	Ask the patient to rate each psychosocial goal.
Step 9	Re-cap both surgical and psychosocial goals with importance ratings. 9.1. Show the patient the PEGASUS sheet and ask if there is anything they want to add.
Step 10	Instruct the patient to take the completed PEGASUS sheet into their consultation with the surgeon (part 2) and explain why this is important. 10.1. Take 2 photocopies (3 copies in all) of the PEGASUS sheet. Place a copy in the notes. Give a copy to the patient. 10.2. Is there anything else you want to ask?
Stage 2: the meeting between the surgeon and patient^b^	
Step 1	Look at the completed PEGASUS sheet which contains your patients surgical and psychosocial goals and use it to facilitate a consultation that is focussed around the patients individual goals
Step 2	Rank (from 0 to 10) the probability of achieving each surgical goals and write this down on the PEGASUS sheet
Step 3	Reflect with the patient on the extent to which psychosocial goals are likely to follow. (Do not rank the probability of achieving psychosocial goals)^c^
Step 4	Use the PEGASUS sheet along with your expert knowledge and experience of breast reconstruction surgery to identify if the patient has realistic expectations and then, if necessary, take steps to address and manage any that you consider to be unrealistic. Place your PEGASUS sheet back in the patient’s notes and ensure that the patient has her copy to take away
Step 5	At the patient’s follow-up appointment (post-surgery) use the PEGASUS sheet once again to assist the discussion and reflect on the extent to which the surgical goals have been achieved

^a^Action: What you as the PEGASUS coach need to do

^b^Action: What you as the surgeon need to do

^c^Whilst surgeons are well placed to comment on the probability of achieving surgical goals, they do not know how these are likely to impact for any given individual. Interestingly, the National Audit report page 36 makes a similar distinction ‘These results (on sexual satisfaction) are likely to reflect many issues that cannot be dealt with by the surgical team’

In intervention stage 1, the patient meets with a PEGASUS coach. In intervention stage 2, the same patient meets with the surgeon managing their care who uses the information elicited in intervention stage 1 to frame the discussion of surgery. Appointments can be same day (for women travelling longer distances) to 2 weeks as per usual care pathway. Because the intervention has an output, i.e. is recorded on the PEGASUS sheet, progress can be regularly reviewed to check that the steps in each stage of PEGASUS are being followed as intended. A detailed manual and video of an example intervention are available to make this detail explicit (contact author). Finally, sessions are recorded for review to ensure fidelity of the intervention during the evaluation phase.

## Discussion and Conclusion

The COM-B model provides a logical and systematic model to describe the design of a complex intervention for supporting shared decision-making in breast reconstruction. We made use of a substantial data source (National Mastectomy and Breast Reconstruction Audit) to identify a problem (patient dissatisfaction after breast reconstruction) followed by the design of an intervention to improve these patient outcomes which was developed and is undergoing systematic evaluation in line with the MRC model for complex interventions. Whilst ‘patient dissatisfaction’ can be criticised for being an outcome rather than a behaviour, it is closely associated with behaviours such as avoidance of intimacy, changes in lifestyle choices (type of clothing) and requests for additional surgery; thus it is a key patient reported factor in assessing the utility of surgery and was readily available via the audit [1].

This paper describes a retrospective fit of the COM-B model to provide explicit detail of each step in the intervention design. There are a number of criticisms that can be made of this. Clearly, a prospective approach would have been preferable but the model was not in common use in the early stages of developing PEGASUS and we note the precedent in use of retrospectively coding BCTTv1 as part of the Theories and Techniques of Behaviour Change Project (www.ucl.ac.uk/health-psychology/research/TaT). Even used prospectively, the COM-B model has some difficulties. It assumes that problem behaviour is well understood and has been clearly identified and defined. In our study, this was facilitated via a robust data source and the intervention designed as a response to this. However, in considering requests to use PEGASUS with other patient groups, we have become aware of the problems that can arise where intervention development precedes assessment and understanding of the underlying problem. We would argue that shared decision-making should be viewed as a means to an end (increased satisfaction with outcome and the decision made) and not the goal of an intervention per se. Secondly, although COM-B is a systematic approach to behaviour change, there are a number of stages when decisions about intervention become subjective. For example, in choosing the sources of behaviour which seem to have the greatest potential utility and are the easiest to implement, judgments depend on the quality of information that can be accessed (for example from focus groups) or benefit from experience of researchers working within given systems or with particular client groups. The same criticism can be made about the objectiveness of selection of intervention functions, which are generally achieved via consensus as in this study. However, COM-B does allow greater rigour to be applied to the design of content of an intervention and thus rebalances the prevailing mismatch between intervention design and evaluation.

Our selection of shared decision-making and attempt to operationalise this can be criticised. Shared decision-making is a concept with many different definitions, and there is room for disagreement about the behaviours we selected to underpin this approach. However, we do have some preliminary evidence from the feasibility study that supports this choice [20]. Feedback was extremely positive—women found that completing PEGASUS alongside a discussion with a specially trained health professional helped them prepare for the surgical consultation and increased their trust in their surgeon. Staff reported that PEGASUS facilitated patient-centred discussions and informed the decisions made about potential surgery. PEGASUS is now undergoing a multicentre controlled trial to establish its effectiveness with a large patient group when compared with usual care and its potential impact on improving satisfaction with decision-making and outcome in breast reconstruction surgery [23]. Whilst data analysis in ongoing, early indications are that patients in the intervention group report a more positive experience in terms of organising their priorities at a time when they feel ‘overwhelmed’ by their diagnosis and express lower post-operative regret about the decisions they have made. Health professionals are similarly positive about the structure that PEGASUS provides for ensuring shared decision-making. If successful, we anticipate that it will have utility in other clinical settings.
